# Extensive fragmentation of the X chromosome in the bed bug *Cimex lectularius* Linnaeus, 1758 (Heteroptera, Cimicidae): a survey across Europe

**DOI:** 10.3897/CompCytogen.v7i4.6012

**Published:** 2013-10-03

**Authors:** David Sadílek, František Šťáhlavský, Jitka Vilímová, Jan Zima

**Affiliations:** 1 Charles University in Prague, Faculty of Science, Department of Zoology, Viničná 7, CZ-12844 Praha, Czech Republic; 2 Institute of Vertebrate Biology, Academy of Sciences of the Czech Republic, Květná 8, CZ-60365 Brno, Czech Republic

**Keywords:** *Cimex lectularius*, *Cimex pipistrelli*, cytogenetics, chromosome number variation, X chromosome

## Abstract

Variation in the number of chromosomes was revealed in 61 samples of *Cimex lectularius* Linnaeus, 1758 from the Czech Republic and other European countries, hosted on *Myotis* Kaup, 1829 (4) and *Homo sapiens* Linnaeus, 1758 (57). The karyotype of all the specimens of *Cimex lectularius* analysed contained 26 autosomes and a varying number of the sex chromosomes. The number of sex chromosomes showed extensive variation, and up to 20 fragments were recorded. Altogether, 12 distinct karyotypes were distinguished. The male karyotypes consisted of 29, 30, 31, 32, 33, 34, 35, 36, 37, 40, 42 and 47 chromosomes. The females usually exhibited the number of chromosomes which was complementary to the number established in the males from the same sample. However, 11 polymorphic samples were revealed in which the karyotypes of females and males were not complementary each other. The complement with 2n = 26+X_1_X_2_Y was found in 44% of the specimens and 57,4% samples of bed bugs studied. The karyotypes with higher chromosome numbers as well as individuals with chromosomal mosaics were usually found within the samples exhibiting particularly extensive variation between individuals, and such complements were not found within samples contaning a few or single specimen. The occurrence of chromosomal mosaics with the karyotype constitution varying between cells of single individual was observed in five specimens (4.3%) from five samples. We assume that polymorphism caused by fragmentation of the X chromosome may result in meiotic problems and non-disjunction can produce unbalanced gametes and result in lowered fitness of individuals carrying higher numbers of the X chromosome fragments. This effect should be apparently enhanced with the increasing number of the fragments and this may be the reason for the observed distribution pattern of individual karyotypes in the studied samples and the rarity of individuals with extremely high chromosome numbers. The assumed lowering of the fitness of individuals carrying higher numbers of the X chromosome fragments could affect population dynamics of variable populations.

## Introduction

The genus *Cimex* Linnaeus, 1758 is the best known taxon of the family Cimicidae (Heteroptera) which contains up to 110 described species of haematophagous ectoparasites exploiting mostly bats and birds as hosts (Usinger 1966, Péricart 1996, Henry 2009). The human bed bug *Cimex lectularius* Linnaeus, 1758, one of the two most important *Cimex* species parasiting on humans, is a temporal haematophagous ectoparasite usually found in human dwellings and bat roosts as well as on domestic and synanthropic vertebrates (Usinger 1966, Schuh and Slater 1995, Reinhardt and Siva-Jothy 2007). The bed bug was practically eradicated by a mass use of DDT in the 1940s and 1950s but it has re-started new expansion in all developed countries of the Temperate Zone during the last ten years (Hwang et al. 2005, Romero et al. 2007). Due to its reemerging history as a human pest the species has been intensively studied (e.g. Reinhardt and Siva-Jothy 2007, Szalanski et al. 2008, Balvín et al. 2012).

Karyotypic variation within the family Cimicidae and the genus *Cimex* is believed to be frequently related to the sex chromosomes. The XY sex determination system was proposed as ancestral in 53 species of cimicids that have been studied cytogenetically so far, and the diploid number in male complements varies from 2n=10 to 47, with the modal number of 31 (Ueshima 1979, Kuznetsova et al. 2011). Systems including multiple sex chromosomes were revealed in various species. The X_1_X_2_Y constitution prevails but several species showed karyotypes with three, four or even more X chromosomes (Ryckman and Ueshima 1964, Ueshima 1966, 1979, Manna 1984, Grozeva and Nokkala 2002, Poggio et al. 2009, Simov et al. 2006, Kuznetsova et al. 2011). Intraspecific variation in the number of sex chromosomes was also reported in two species of the genus *Paracimex* Kiritshenko, 1913 parasiting in birds (Ueshima 1968).

The bed bug, *Cimex lectularius*, shows combination of unusual cytogenetic characteristics, partly common for all Heteroptera. The chromosomes are holokinetic, with completely achiasmatic male meiosis of collochore type and inverted meiosis of the sex chromosomes. A particularly remarkable feature is numerical variation in the number of the sex chromosomes. The standard karyotype of the bed bug contains 26 autosomes and a varying number of supernumerary chromosomes which is supposed to originate after fragmentation of the X chromosome (e.g. Ueshima 1966, Grozeva et al. 2010). Variation in the chromosome number in the bed bug karyotype was first reported by Darlington (1939) and Slack (1939) from Great Britain. Darlington (1939) distinguished in natural populations 13 karyotypes containing two up to 14 X chromosomes. Most of the specimens examined possessed complements with higher chromosomal number. Slack (1939) revealed the presence of 13 karyotypes with the number of the X chromosomes varying between three and 15. Ueshima (1966, 1967) studied nine samples of bed bugs collected in various continents and he was able to recognize five karyotypes with the number of the X chromosomes varying from two to nine. Grozeva et al. (2010, 2011) examined small samples of bed bugs originating from Russia (St Petersburg) and Bulgaria (Sofia) and recorded the standard karyotype only (2n=26+X_1_X_2_Y).

The related species *Cimex pipistrelli* Jenyns, 1839 is known as an obligate parasite of bats which may share its hosts with *Cimex lectularius*. The karyotype of *Cimex pipistrelli* is similar to the standard complement of *Cimex lectularius* but contains a higher number of autosomes (2n=28+X_1_X_2_Y; Ueshima 1966). No variation in the chromosome number has been recorded in this species.

The recent expansion is a reason why cytogenetic analysis of this species starts to be more important in respect of recent findings indicating that karyotypic divergences could have evolved faster than DNA sequences (e.g. Britton-Davidian et al. 2007, Horn et al. 2012). This is another piece of evidence that initial evolution at the genomic, karyotypic and organismal level can proceed rather independently, as is apparently the case of the bed bug. The intraspecific karyotypic variation may be associated with segregation irregularities resulting in possible lowering of the fitness. Research of this variation can thus provide more understanding of reproductive biology and population dynamics of the bed bug.

This study reports cytogenetic findings in *Cimex lectularius* and *Cimex pipistrelli* based on large samples of studied individuals from the Czech Republic and other European countries. We aim to investigate karyotypic variation reported previously in the bedbug and to obtain data revealing possible temporal and geographic pattern of this variation. Another goal of this study is to contribute to better understanding of the mechanisms underlying this variability.

## Material and methods

**Figure 1.**
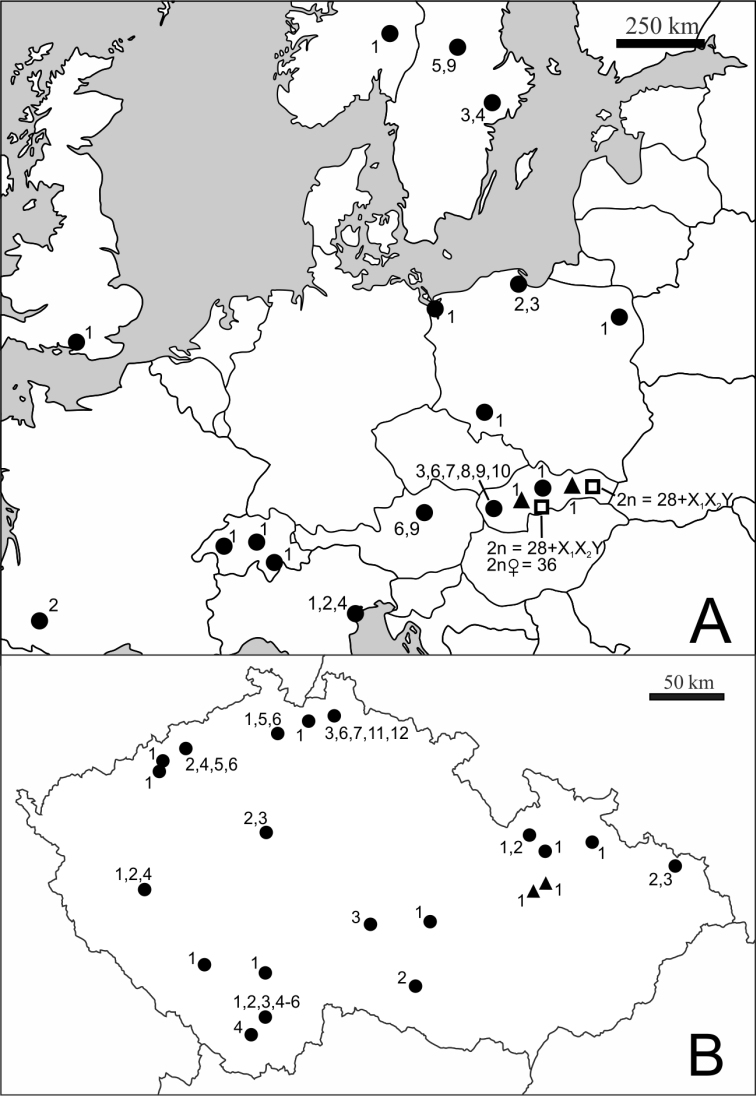
Geographical distribution of the sites studied. **A** Samples of *Cimex lectularius* and *Cimex pipistrelli* from Europe **B** Samples of *Cimex lectularius* from Czech Republic. ● *Cimex lectularius*, human habitats, ▲ *Cimex lectularius*, bat roosts, □ *Cimex pipistrelli*. Numbers refer to karyotypes 1–12 described in Results.

The studied specimens of *Cimex lectularius* and *Cimex pipistrelli* were collected from bat roosts and human dwellings in 2010–2012 (Fig. 1). The karyotype was determined in 116 specimens of *Cimex lectularius* from 61 localities within 10 European countries and in five specimens of *Cimex pipistrelli* from two localities in Slovakia. The live individuals of synantropic bed bugs from humans were mostly collected by pest exterminators in flats, hotels and hostels. The studied samples originated from individual collecting sites which were localized with varying levels of precision, particularly in the synathropic habitats (flat, house, town, city) depending on information available from the collectors. Individual sites within a single city are differentiated by numerals (e.g., Prague 1, Prague 2). Bugs identified as *Cimex lectularius* were also collected at four sites of bat roosts in the Czech Republic and Slovakia. The complete list of the collecting sites is shown in Table 1.

**Table 1.** The list of the collecting sites and a summary of primary results. A = Austria, CH = Switzerland, CZ = Czech Republic, F = France, GB = Great Britain, I = Italy, N = Norway, PL = Poland, S = Sweden, SK = Slovakia. Specimens: left column males, right column females. Designation of the type of karyotype in the last column is the same as in the text and Table 2.

**Sample Code**	**Country**	**Locality**	**Specimens**		**Karyotype**
*Cimex pipistrelli*			♂	♀	
190	SK	Hontianske Nemce	1	2	see text
191	SK	Ľubovec	2		see text
*Cimex lectularius*					
Host: *Myotis myotis* (Borkhausen, 1797), *Myotis emarginatus* (E. Geoffroy, 1806)					
417	CZ	Bílá Lhota		1	1
418	CZ	Moravičany	1		1
421	SK	Krásnohorské Podhradie	2		1
423	SK	Hosťovce	2	1	1
*Cimex lectularius*					
Host: *Homo sapiens*					
609	CZ	Bruntál	1		1
610	CZ	Plzeň (1)	2	1	1
612	CZ	Chomutov – Dřínovská	1	1	1
613	CZ	Liberec (1) – Krejčího	2	1	3, 6
614	CZ	Liberec (2)- Krejčího	3		7, 11, 12
615	CZ	Jirkov - Na Borku	1	1	1
617	CZ	Štědrákova Lhota	1	1	1,2
618	CZ	Stráž pod Ralskem	1		1
619	CZ	Bohumín – Studentská	3		2,3
621	CZ	Plzeň (2) – Na Vinicích		2	1
623	CZ	Šumperk	1	1	1
624	CZ	Plzeň (3) – Na Slovanech	1	1	1
625	CZ	Plzeň (4) – Na Slovanech	2	1	1
629	CZ	České Budějovice (1) – Puklicova		1	3
632	CZ	Janov	1	2	2, 4, 5, 6
633	CZ	Jaroměřice nad Rokytnou	1		2
634	CZ	Plzeň (5)	1		2
640	CZ	Plzeň (6) – Na Slovanech	2		1
642	CZ	Praha (1)	2		2
643	CZ	Praha (2)	1		3
644	CZ	České Budějovice (2)		2	1
645	CZ	České Budějovice (3) - Okružní		1	3
647	CZ	Praha (3)		1	2
648	CZ	Praha (4)	3		2
657	CZ	Plzeň (7)	2		1, 4
658	CZ	Humpolec	2	1	3
659	CZ	Praha (5) – Křížíkova		1	2
661	CZ	Česká Lípa – Svárovská	3	1	1, 5, 6
662	CZ	České Budějovice (4) – Netolická	1	1	2, 4-6
665	CZ	Chvalšiny		2	4
667	CZ	Týn nad Vltavou – Hlinecká	1		1
668	CZ	České Budějovice (5) – J. Bendy	1		1
669	CZ	Strakonice – Bezděkovská	1		1
670	CZ	České Budějovice (6) – M. Chlajna	2		1
671	CZ	Žďár nad Sázavou	1		1
707	SK	Banská Bystrica	2		1
708	SK	Trnava	5	1	3, 6, 7, 8, 9, 10
719	GB	Brighton		1	1
720	A	Melk	1	2	6, 9
732	CH	Luzern	1		1
737	CH	-		1	1
745	CH	Fribourg – Rue de l´Hôpital	1	1	1
750	I	Mestre	1	2	2
751	I	Venezia (1)	1		1
752	I	Venezia (2)	2	1	1, 2
753	I	Venezia (3)	1		1, 4
789	N	Ottestad	1		1
795	S	Borlänge (1)	2		5
796	S	Borlänge (2)	1	1	5, 9
798	S	Stockholm – Vårber	1	2	3, 4
817	F	Aire/Adour		2	2
831	PL	Świnoujscie		1	1
838	PL	Gdansk (1)	1		2
840	PL	Gdansk (2)	2	1	2, 3
843	PL	Wroclav – Grabiszynska		1	1
844	PL	Białystok (1)	1		1
845	PL	Białystok (2)	1		1

The chromosome preparations were made from gonads or midgut using the spreading technique described by Traut (1976) modified after Šťáhlavský and Král (2004). Briefly, the tissues were dissected and hypotonised in 0.075 M KCl solution for 25 minutes and then fixed in glacial acetic acid:methanol (1:3) for 15–25 minutes. The fixed material was suspended in a drop of 60% acetic acid on a microscope slide and the slide was placed on a warm histological plate (temperature 40–45°C). The drop was than moved on the slide until it evaporated. The chromosome preparations were stained in a 5% Giemsa solution in Sörensen phosphate buffer (pH = 6.8) for 30 minutes. The chromosome slides were examined with the use of the Olympus Provis AX 70 microscope and selected cells and stages of division were documented by the digital imaging system Olympus DP 72 and software QuickPHOTO CAMERA 2.3. The diploid chromosome complements of males were described by the formula 2n=26+X_1-n_Y where n stands for the additional X chromosomes. The corresponding karyotypes of females were characterized by the formula 2n=26+2X_1-n_.

After withdrawing of tissues for cytogenetic methods, the material was preserved in 96% ethanol and used in parallel molecular studies. Their results have approved the original specimens determination according to morphological characters (Balvín et al. 2012, 2013). The material is deposited in collections of the Department of Zoology, Charles University in Prague.

## Results

The karyotype of all the specimens of *Cimex lectularius* analysed contained 26 autosomes and a varying number of the sex chromosomes. The relative length of chromosomes in the complement was successively diminishing from 5.3 to 1.7%. No distinct size groups of chromosomes could be differentiated; however, the largest and the smallest autosomal pair could be usually recognized according to their size. The original sex chromosomes X_1_X_2_Y were medium-sized whereas their supposed fragments occurring in the karyotypes with higher chromosome numbers were the smallest elements of the set.

In the samples of *Cimex lectularius* studied, 12 distinct karyotypes were differentiated (Table 2). These karyotypes were distinguished according to the varying diploid chromosome number (2n=29–37, 40, 42, 47 in the male complement) and the varying number of the X chromosomes (2–20).

**Table 2.** The distribution of samples studied in individual karyotypes characterized in the text. A = Austria, CH = Switzerland, CZ = Czech Republic, F = France, GB = Great Britain, I = Italy, N = Norway, PL = Poland, S = Sweden, SK = Slovakia. Single female possessing the odd number of chromosomes is not included.

**Karyotype**	**2n**	**Sex chromosomes**	**No. of samples**	**<br/> %**	**No. of specimens**	**<br/> %**	**Country**
1	29	2XY	35	57.4	51	44.0	CZ, GB, CH, I, N, PL, SK
2	30	3XY	15	24.6	24	20.7	CZ, F, I, PL
3	31	4XY	9	14.8	13	11.2	CZ, S, SK
4	32	5XY	4	6.6	5	4.3	CZ, S
5	33	6XY	3	4.9	5	4.3	CZ, S
6	34	7XY	3	4.9	3	2.5	A, CZ, SK
7	35	8XY	2	3.3	2	1.7	CZ, SK
8	36	9XY	1	1.6	1	0.9	SK
9	37	10XY	3	4.9	3	2.5	A, S, SK
10	40	13XY	1	1.6	1	0.9	SK
11	42	15XY	1	1.6	1	0.9	CZ
12	47	20XY	1	1.6	1	0.9	CZ
mosaic	-	-	5	8.2	5	4.3	A, CZ, I, SK

The identical karyotype was found in all the specimens studied in 46 monomorphic samples, whereas karyotype differences were recorded between individuals in 15 polymorphic samples. We should note, however, that about half of the studied samples (26) consisted of a single specimen only. The results recorded in individual collecting sites are summarized in Table 1.

**Figure 2.**
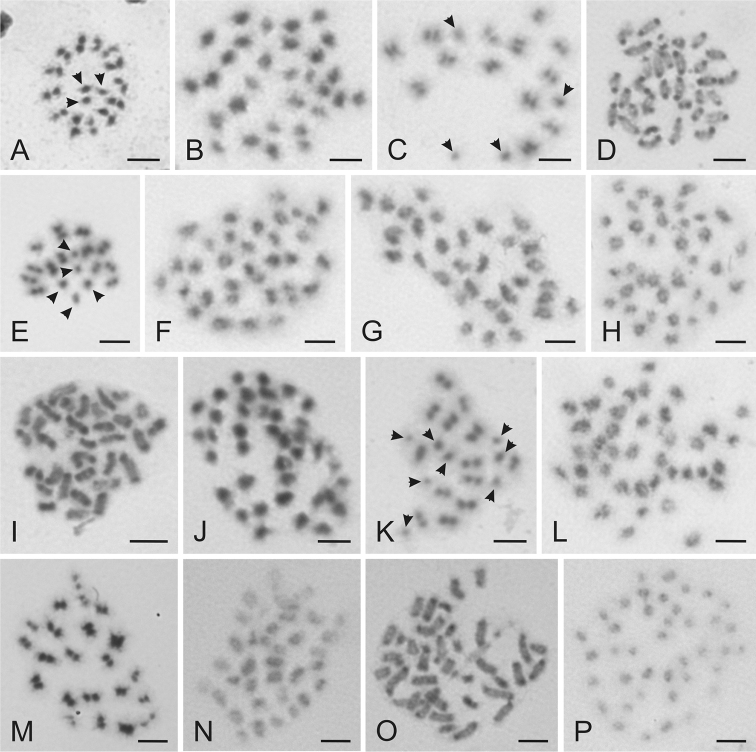
Examples of chromosomes of *Cimex lectularius* from various stages of cell division stained with Giemsa. **A** Metaphase II ♂, 2n=29 **B** Mitotic metaphase ♀, 2n=30 **C** Metaphase II ♂, 2n=30 **D** Mitotic prometaphase ♀, 2n=32 **E** Metaphase II ♂, 2n=31 **F** Mitotic metaphase ♀, 2n=34 **G** Mitotic metaphase ♂, 2n=32 **H** Mitotic metaphase ♀, 2n=36 **I** Mitotic prometaphase ♂, 2n=33 **J** Mitotic metaphase ♀, 2n=38 **K** Metaphase II ♂, 2n=34 **L** Mitotic metaphase ♀, 2n=40 **M** Metaphase I ♂, 2n=35 **N** Mitotic metaphase ♂, 2n=36 **O** Mitotic prometaphase ♂, 2n=37 **P** Mitotic metaphase ♂, 2n=40. Arrows indicate sex chromosomes. Bar = 5 μm.

The most common karyotype 1 was characterized by the standard complements with two X chromosomes; 2n=29 in males (2n=26+X_1_X_2_Y) and 2n=30 in females (2n=26+X_1_X_1_X_2_X_2_) (Fig. 2a, b). This complement was found in 51 specimens (33 males and 18 females) and in 31 monomorphic and four polymorphic samples. Seven monomorphous samples of this karyotype included females only. The monomorphic samples from synanthropic habitats were collected in the Czech Republic, Great Britain, Italy, Norway, Poland, Slovakia and Switzerland. This karyotype was further recorded in some individuals from the polymorphic samples collected in the Czech Republic and Italy and in the all samples of *Cimex lectularius* collected in bat roosts (Fig. 1).

Karyotype 2 included complements with three X chromosomes; 2n=30 in males (2n=26+X_1-3_Y) and 2n=32 in females (2n=26+2X_1-3_) (Fig. 2c, d). This chromosome constitution was recognized in 24 specimens (15 males and 9 females) from 15 samples. The karyotype was recorded in both the monomorphic and polymorphic samples. The monomorphic samples from synanthropic habitats in the Czech Republic and Poland included males only, the sample from Italy included males and females, and other samples from the Czech Republic and France included females only. This karyotype was further found in polymorphic samples from the Czech Republic, Italy and Poland.

Karyotype 3 included complements with four X chromosomes; 2n=31 in males (2n=26+X_1-4_Y) and 2n=34 in females (2n=26+2X_1-4_) (Fig. 2e, f). This complement was found in 13 specimens (8 males and 5 females) from nine samples. The karyotype was recorded in monomorphic samples from the synathropic habitats collected in the Czech Republic and in polymorphic samples from the Czech Republic, Slovakia and Sweden.

Karyotype 4 included complements with five X chromosomes; 2n=32 in males (2n=26+X_1-5_Y) and 2n=36 in females (2n=26+2X_1-5_) (Fig. 2g, h). It was found in five specimens (2 males and 3 females) from four samples. This complement was recorded in a monomorphic sample from the Czech Republic and in polymorphic samples from the Czech Republic and Sweden.

Karyotype 5 included complements with six X chromosomes; 2n=33 in males (2n=26+X_1-6_Y) and 2n=38 in females (2n=26+2X_1-6_) (Fig. 2i, j) and it was found in five specimens (4 males and 1 female) from three samples. This complement was identified in a single monomorphic sample including two males collected in Sweden and in polymorphic samples from the Czech Republic and Sweden.

Karyotype 6 included complements with seven X chromosomes; 2n=34 in males (2n=26+X_1-7_Y) and 2n=40 in females (2n=26+2X_1-7_) (Fig. 2k, l) and it was found in three specimens (1 male and 2 females) from three polymorphic samples collected in Austria and the Czech Republic.

Karyotype 7 included a complement with eight X chromosomes and 2n=35 in males (2n=26+X_1-8_Y) (Fig. 2m) and it was found in two male specimens collected in polymorphic samples from two sites in the Czech Republic and Slovakia, respectively.

Karyotype 8 included a complement with nine X chromosomes and 2n=36 in males (2n=26+X_1-9_Y) (Fig. 2n) and it was recorded in a single male collected in the Slovakia.

Karyotype 9 included a complement with ten X chromosomes and 2n=37 in males (2n=26+X_1-10_Y) (Fig. 2o) and it was recorded in three males collected in the Austria, Slovakia and Sweden.

Karyotype 10 included a complement with 13 X chromosomes and 2n=40 in males (2n=26+X_1-13_Y) (Fig. 2p). This karyotype was identified in a single male collected in Slovakia.

**Figure 3.**
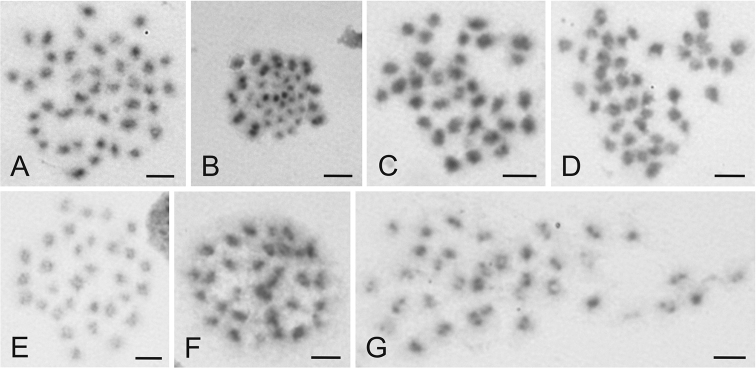
Examples of chromosomes of *Cimex lectularius* (**A–D**) and *Cimex pipistrelli* (**E-G**) from various stages of cell division stained with Giemsa. **A** Mitotic metaphase ♂, 2n=42 **B** Metaphase II ♂, 2n=47 **C** Mitotic metaphase ♀, 2n=33 **D** Mitotic metaphase ♀, 2n=43 **E** Mitotic metaphase ♂, 2n=31 **F** Mitotic metaphase ♀, 2n=32 **G** Mitotic metaphase ♀, 2n=36. For more details see text. Bar = 5 μm.

Karyotype 11 included a complement with 15 X chromosomes and 2n=42 in males (2n=26+X_1-15_Y) (Fig. 3a). This karyotype was identified in a single male collected in the Czech Republic.

Karyotype 12 included complements with 20 X chromosomes and 2n=47 in males (2n=26+X_1-20_Y) (Fig. 3b). This complement was identified in a single male from the Czech Republic.

The females exhibited the number of chromosomes which was usually complementary to the number established in the males from the same sample. However, 11 polymorphic samples were revealed in which the karyotypes of females and males were not complementary one another. Two females showing karyotypes with odd numbers of X chromosomes (7 and 17; 2n=33 and 43, respectively) were recorded (Fig. 3c, d).

The occurrence of chromosomal mosaics with the karyotype constitution varying between cells of single individual was observed in five specimens (2 males and 3 females) from five samples. A female from Slovakia (Trnava) had two karyotypically different cell types. The complement with 14 X chromosome fragments (2n=40) was found in mesenteron cells, whereas 17 X chromosome fragments (2n=43) were observed in germinal cells from ovarium. In other individuals showing mosaic, variation was recorded between germinal cells derived from gonads only. In a single female from Austria (Melk), the karyotypes with 12 and 14 X chromosome fragments were recorded (2n=38 and 40, respectively). A male from Janov in the Czech Republic showed cells with six or seven X fragments (2n=33 and 34, respectively). A similar mosaic constitution was recorded in a female from České Budějovice (4) (2n=36 and 40, respectively). Mosaicism with two and five X chromosome fragments was also revealed in a male from Italy (Venezia 3) (2n=29 and 32, respectively).

The karyotypes with higher chromosome numbers as well as individuals with chromosomal mosaic were usually found within the samples exhibiting particularly extensive variation between individuals. A sample from Liberec contained two males with karyotypes 2n=26+X_1-4_Y and a single female with 2n=26+2X_1-14_. The other sample from Liberec, collected in another flat in the same house, included three males with distinctly different karyotypes (2=26+X_1-8_Y, 2n=26+X_1-15_Y, 2n=26+X_1-20_Y). The Trnava sample contained five males with different karyotypes (2n=26+X_1-4_Y, 2n=26+X_1-8_Y, 2n=26+X_1-9_Y, 2n=26+X_1-10_Y, 2n=26+X_1-13_Y) and a female showing a mosaic karyotype with different chromosomal numbers observed in both examined tissues. The Melk sample included a male with 2n=26+X_1-10_Y, and two females, one with 2n=26+2X_1-7_ and another with a mosaic karyotype constitution 2n=26+2X_1-12/14_.

The karyotype of *Cimex pipistrelli* included 28 autosomes and the sex chromosome trivalent X_1_X_2_Y (males 2n=28+X_1-2_Y=31, females 2n=28+2X_1-2_=32; Fig. 3e, f). This complement was found in four specimens examined. The complement of a female from Slovakia (Hontianske Nemce) contained eight X chromosomes (2n=28+2X_1-4_=36; Fig. 3g).

## Discussion

Our data confirm considerable variation in the karyotype of the bed bug and further extend its range (Table 3). The distribution of the karyotypes in various Czech and European localities appeared random, and did not show any consistent geographic pattern. Therefore, no reliable information concerning the historical or current dispersal of bed bugs can be derived.

**Table 3.** A synopsis of known karyotypes in*Cimex lectularius*. References: 1 - Darlington 1939, 2 - Slack 1939, 3 - Ueshima 1966, 4 - Grozeva et al. 2010, 5 - Grozeva et al. 2011. BG = Bulgaria, ET = Egypt, J = Japan, MEX = Mexico, RUS = Russia, USA = United States of America. See Tables 1 and 2 for explanation of other countries abbreviations. The samples reported in this study in bold.

**Karyotype**	**X, Y**	**2n**	**Country**	**References**
1	2XY	29	**CH**, **CZ**, BG, F, **GB**, **I**, J, MEX, **N**, **PL**, RUS, **SK**, USA	1, 3, 4, 5, this study
2	3XY	30	**CZ**, **F**, GB, **I**, **PL**	1, 2, this study
3	4XY	31	**CZ**, GB, **S**, **SK**	1, 2, this study
4	5XY	32	**CZ**, GB, **I**, **PL**, **S**	1, 2, this study
5	6XY	33	**CZ**, ET, GB, **PL**, **S**, USA	1, 2, 3, this study
6	7XY	34	**A**, **CZ**, GB, **PL**, **SK**, USA	1, 2, 3, this study
7	8XY	35	**CZ**, GB, **SK**, USA	1, 2, 3, this study
8	9XY	36	GB, **SK**, USA	1, 2, 3, this study
9	10XY	37	**A**, GB, **S**, **SK**	1, 2, this study
10	11XY	38	GB	1, 2
11	12XY	39	GB	1, 2
12	13XY	40	GB, **SK**	1, 2, this study
13	14XY	41	GB	1, 2
14	15XY	42	**CZ**, GB	2, this study
15	20XY	47	**CZ**	this study

We have obtained certain findings that are at variance with the previously published results. The distribution pattern of incidence of the X chromosome fragments reported by Darlington (1939) is different from that revealed in our study. Darlington (1939) recorded mostly individuals with higher chromosome numbers and the numbers of the X chromosomes higher then six prevailed in his samples (23 specimens out of the 25 examined ones). In our study, individuals with lower numbers clearly prevailed. In the samples examined, 89 out of the 116 specimens studied had less then five X chromosomes in their complements and the karyotype containing only two X chromosomes was recorded in approximately a half of the specimens studied. This pattern is fairly congruent with data obtained by Slack (1939), Ueshima (1966, 1967) and Grozeva et al. (2010, 2011). We have not recorded some of the karyotypes reported by Darlington (1939) and Slack (1939) in the studied European samples. This absence is apparently related to random sampling. On the other hand, our study has revealed the highest known chromosome number in the male bedbug karyotype with 47 chromosomes (2n=26+X_1-20_Y). This represents the highest X chromosome number recorded within Cimicidae, Heteroptera and probably also Insecta.

The results obtained in *Cimex pipistrelli* confirm the previously published data (Ueshima 1966) in respect of the standard karyotype but the finding in a single female indicate that variation in the number of the X chromosomes may rarely occur also in this species.

There are various possible explanations of the origin of extensive variation in the chromosome number in the karyotypes of bed bugs. The elements responsible for numerical variation in bed bugs could belong to a specific chromosomal type known in other heteropterans. In 14 families of this order, a special pair of chromosomes occurs called the m-chromosomes (e.g. Ueshima 1979). The size of these chromosomes is distinctly smaller than that of other chromosomes, and their meiotic behaviour is unusual. The origin and significance of these elements remain unknown (Ueshima 1979, Ituarte and Papeschi 2004, Rebagliati et al. 2005, Papeschi and Bressa 2006, Kuznetsova et al. 2011). It is quite improbable that the supernumerary chromosomes producing the numerical variation between karyotypes of bed bugs are related to the m-chromosomes. The small supernumerary elements in the bed bug complement are rarely negatively heteropycnotic and they enter the reductional division as late as in the metaphase II, similarly as typical sex chromosomes. There is no evidence of the presence of the m-chromosomes in karyotypes of bed bugs.

B chromosomes were reported in species from various bug families including Cimicidae (Ueshima 1966, Grozeva and Nokkala 2001, Pérez et al. 2004, Panzera et al. 2010). The characteristic of the B chromosomes is different compared to the supernumerary elements from the bed bug complements. These additional chromosomal fragments are not distributed randomly, they are mainly isochromatic and they do not show any signs of heterochromatinization.

Therefore, the most plausible explanation of the origin of the supernumerary elements in the bed bug complements remains fragmentation of the X chromosome. This mechanism produces a complicated system of multiple sex chromosomes and it was proposed already in previously published papers (see Ueshima 1966, 1979 for review). This explanation was supported by the observed behaviour of the fragments in meiosis and also by comparisons with other related species of the genus *Cimex*. Similar systems have been commonly found in some other heteropteran species but the extent of variation is usually limited (Papeschi and Bressa 2006). In the bed bug, the supposedly original fragmentation of the X chromosome into two segments (X_1_X_2_) has already become widely fixed in the extant populations. However, it is not sure that the assumed original fission resulted always in the formation of the same fragments. Similarly, the nature of subsequent fissions producing successively other fragments is not clear and may vary. The karyotypes of females with higher numbers of the X chromosome fragments could be heterozygous with varying constitution of fragments derived from parents. This possible variation cannot be evidenced with the use of classical cytogenetic techniques and molecular approach should be employed in clarifying this question.

The causes of the origin and maintenance of extensive fragmentation of the X chromosome of bed bugs remain unclear. Populations of bedbugs have been exposed to various insecticides all over the world for decades (Romero et al. 2007, Weeks et al. 2010). Potential mutagenetic effects of these toxic substances might have increased the rate of chromosomal rearrangements in bed bugs. The increased incidence of the X chromosome fragments in synanthropic populations seems to support this explanation as well as the absence of the multiple X chromosome fragments found in populations parasiting bats. However, variation resulting from this mechanism was recorded in the related species *Cimex pipistrelli* which does not occur in man and, rarely, also in other species of the family Cimicidae (genus *Paracimex*) not related to humans (Ueshima 1968).

We found an extraordinarily wide extent of karyotype variation between specimens in a few population samples only. We assume that this extreme variation might result from random mixing of individuals of different origin at a single site. Mating between geographically unrelated individuals can easily be imagined in a parasite such as the bed bug transmitted by migrating people. However, it is difficult to explain why these highly variable samples usually included specimens with an extreme karyotype constitution and the highest numbers of the X chromosome fragments. It is obvious that mating of parents with different karyotypes can produce great variety of recombinant complements in offspring, particularly in females. Variation in the number of chromosome fragments may be associated with abnormalities occurring in chromosome segregation during the cell division. The regular course of meiosis in the bed bug may be influenced by the holokinetic nature of chromosomes, completely achiasmatic male meiosis and inverted meiosis of the sex chromosomes. Slack (1939) and Ueshima (1964, 1966, 1967) recorded varying chromosome numbers among germ cells of single individual. We found similar mosaics in five specimens examined. The irregular meiotic division or meiotic drive may enhance segregational variation in the chromosome number in progeny as well as mitotic segregation disturbances may contribute to the origin of mosaics in somatic cells. Mating of individuals of different origin and possibly different genetic constitution may initiate and increase the occurrence of segregation problems. We can assume that non-disjunction can produce unbalanced aneuploid gametes and result in lowered fitness of individuals carrying higher numbers of the X chromosome fragments. This effect should be apparently enhanced with the increasing number of the fragments and this may be the reason for the observed distribution pattern of individual karyotypes in the studied samples and the rarity of individuals with extremely high chromosome numbers. On the other hand, meiotic drive could cause preferential transmission of certain karyotype variants to the offspring. Ueshima (1966) investigated experimentally the transmission of different parental karyotypes to hybrids but did not report any evidence of aneuploidy or meiotic drive. We have not found any indication of such abnormalities of the cell division also in our study.

We can only speculate about relationships between the system of transmission of the fragmented sex chromosomes and the unusual features of reproductive biology of bed bugs. The assumed lowering of the fitness of individuals carrying higher numbers of the X chromosome fragments could potentially affect population dynamics of variable populations. It is apparent that more intensive cytogenetic screening combined with data on molecular variation in DNA sequences might shed light to this question.
